# Impact of probe sonication and sulfuric acid pretreatment on graphene exfoliation in water

**DOI:** 10.1038/s41598-023-45874-x

**Published:** 2023-10-28

**Authors:** Meriam Mohammedture, Nitul Rajput, Ana Isabel Perez-Jimenez, Zineb Matouk, Shroq AlZadjali, Monserrat Gutierrez

**Affiliations:** Advanced Materials Research Center, Technology Innovation Institute, PO Box 9639, Masdar City, Abu Dhabi, UAE

**Keywords:** Synthesis of graphene, Characterization and analytical techniques, Nanoscale materials, Two-dimensional materials

## Abstract

Graphene is a 2D material with promising commercial applications due to its physicochemical properties. Producing high-quality graphene economically and at large scales is currently of great interest and demand. Here, the potential of producing high-quality graphene at a large scale via water-phase exfoliation methods is investigated. By altering exfoliation parameters, the production yield of graphene and flake size are evaluated. Pretreatment of the precursor graphite powder using acidic solutions of H_2_SO_4_ at different concentrations is found to increase further the yield and structural quality of the exfoliated graphene flakes. These findings are confirmed through various spectroscopy and surface characterization techniques. Controlling flake size, thickness, and yield are demonstrated via optimization of the sonication process, centrifuge time, and H_2_SO_4_ pretreatment.

## Introduction

The discovery of graphene and its remarkable properties has fascinated the scientific community. Single-layer graphene (SLG) has exceptional mechanical properties, with a Young’s modulus in the order of 1 TPa^1,2^. Additionally, it has high electron mobility in the range of 10,000–50,000 cm^2^ V^−1^ s^−1^^3–5^ and excellent thermal conductivity of above 3000 W/m K^6–8^. These superior properties allow graphene to be used in a range of applications, from drug delivery and chemical sensors to energy absorption and flexible electronics. Recent exciting applications include 3D printing of graphene-reinforced metal matrix composites for tailored properties that involve enhancing the mechanical or electrical properties of the metal matrix^9^. Graphene’s attractive ability to be used as a reinforcing material was also seen in lightweight cement composites where the compressive strength and specific energy absorption were increased significantly^10^. Further, graphene’s unique nanostructure allows it to be used as a robust material platform for quantum computing^11–13^. Another potential application of graphene is in membrane technology for water desalination^14,15^.

Despite having remarkable properties and promising applications, the commercialization of graphene has been rather slow, mainly due to the zero-band gap nature that hinders graphene from being integrated into the semiconductor industry^16^ and the mass-scale production of high-quality graphene in an economical way. In fact, graphene commercialization has been inactive in the past decades due to the issues related to the supply chain in providing high-quality and reliable graphene material. Regardless of all the challenges, a sharp increase in graphene use in commercial products in recent years has been observed and is predicted to grow significantly in the coming years^17,18^. Finding an easy-scalable technique to produce graphene is now, therefore, more important than ever.

A promising route to synthesize large-scale, high-quality monolayer to few layers graphene is via chemical vapor deposition (CVD) approaches^19,20^. On the other hand, a plasma enhanced CVD (PECVD) process could provide monolayer to few layers terminated 3D graphene sheets^21,22^. However, a major issue in using CVD/PECVD was in the production cost and the mass scale production inefficiency in a reasonable amount of time. Recently, few groups have attempted to overcome such problems by inducing roll-to-roll concept for continuous production of graphene^23^. Despite the lab-scale success, commercially, it remains a challenging issue. The production up-scaling of the roll-to-roll (R2R) method is limited by the efficiency in the change of the carbon phase, complexity of infrastructure, need for high temperature, and high vacuum^24,25^. Slow manufacturing and high equipment costs are also considered as the major bottlenecks for the mass production of graphene using the R2R method^26^. In parallel, several graphene exfoliation routes from bulk graphite material are investigated and explored. A few examples are graphene exfoliation using layer engineered method^27^, electrochemical exfoliation process^28,29^, laser ablation method^30^, liquid phase exfoliation method^31,32^, etc. Out of the aforementioned methods, liquid-phase exfoliation (LPE) has been a popular approach for graphene synthesis due to its straightforward nature in processing, synthesis, and inherent capability for mass-scale synthesis at a relativity low cost. LPE works by extracting graphene from graphite by overcoming the interlayer van der Waals force for mass-scale graphene synthesis^33^. This can be done by applying ultrasound to produce a few layer-multilayer graphene sheets at a large scale^34,35^. Additionally, the process is economical and is a potential route to produce high-quality graphene for commercial applications^36^.

Despite recognizing LPE as a low-cost graphene production method, there are still various critical parameters that could control the quality of the exfoliated graphene^35^. The solvent choice^37,38^, sonication mode^36,39^, time, and power could be considered as the critical parameters in this case. By tuning these parameters, properties, including graphene dimension and thickness, can be altered for the desired application. The use of the exfoliation method needs to be better understood so it can be used on a larger scale.

The LPE process still needs to be polished to produce a few layers of graphene characterized by a uniform and intact size. Furthermore, the flake thickness and size distribution of the produced flakes ranges greatly for liquid phase exfoliated graphene. It has been observed that this exfoliation method could give nanosheets of thickness of approximately 1–2 nm^39^. However, mostly, few-layer nanosheets of approximately 1–10 monolayers are regularly produced^40^. Furthermore, the sizes range between 10 nm and 10 µm^41^. Nevertheless, this method allows for an easier scale-up for industrialization, making it highly important to refine. Therefore, by exploring ways to ease the exfoliation process between the layers, such as introducing functional groups^42–44^ in a simple and economical way to reduce the van der Waals force between the layers, the process can become extremely efficient.

The use of pure water as a medium for LPE can be very suitable as using deionized (DI) water helps to avoid any foreign chemical (e.g., surfactant), which could be unfavorable and adds an additional task to remove them during the post-process part. Additionally, synthesizing powder material with inherent morphology of the synthesized material using freeze drying adds further advantage with water solution. The exfoliation of graphene sheets in water is highly desirable as the formation of small active cavitation bubbles in water can stimulate the exfoliation as well as support a stable dispersion^45,46^. The use of water as a liquid phase exfoliation medium using probe sonication with acid pretreatment has not been widely studied. Morton et al.^47^ analysed in-depth the degree of exfoliation, quality, and stability of graphene exfoliation in a mixture of water and ethanol. Tyurnina et al.^48^ focused on ultra-sound assisted LPE technique using a dual frequency approach in pure water. Further assessment to analyse the optimum solution temperature was done by the same group to highlight the importance of temperature and its effects^49^. Gao et al.^50^ designed a novel cyclic ultrasound-assisted exfoliation apparatus that utilized cavitation due to abrupt pressure fluctuations from water bubbles. Kim et al.^51^ utilized a bath sonicator to exfoliate and disperse 2D materials and found that the dissolution of graphite in water would be around 40 °C. These studies have shown the promising nature of using water as a potential solvent for graphene exfoliation and its vast area of optimization.

In this work, we have systematically investigated the graphene exfoliation in deionized water using the probe sonication method. The impact of pretreatment role of bath sonication in acid solution and the effect of centrifuging process has been explored.

## Materials and methods

### Graphene exfoliation

Commercially available high-quality graphite powder (IMERYS: Graphite & Carbon) was used as a source material for the exfoliation. The median particle size reported by the manufacturer was 17 µm. Two different molar concentrations of H_2_SO_4 _: 3 M and 5 M were considered for the pretreatment process. The bath sonication method was carried out at a continuous ultrasonic frequency of 40 kHz for 60 min. The sonication was carried out at room temperature (RT) water. However, during the experiment, the bath temperature was observed to increase, which was then flushed with RT water while pausing the sonication process. The temperature variation was kept within 5–7 °C. Probe Sonicator manufactured by Hielscher Ultrasonics GmbH (UP400St) was implemented to homogenize and exfoliate the graphene flakes. The Sonotrode was made of Titanium with an amplitude ratio of approx. 1:2.55, Ø14 mm (173 mm^2^), and an approximate length of 100 mm. The pulse sonication was performed at 57 W, 40% continuous, and 28% Amplitude parameters.

A centrifuge model Sigma 3-18K manufactured by Sigma Laborzentrifugen GmbH was used to separate the graphene thin layers from the thicker layers. The centrifuging step was carried out at 5000 rpm in a swinging type rotor at a g force of 4332*g and at a temperature of 19 °C for different duration of time. The tool that we have been using is a refrigerated centrifuge type (Sigma 3-18K). The refrigerated centrifuges counteract the temperature fluctuations that could occur during the centrifugation process. Additionally, we have not observed any noticeable fluctuation in the set value in the temperature monitor. Samples (a few drops from the top portion) were collected at different intervals of the centrifuging time of 0 min, 15 min, 30 min, 60 min, 120 min, and 180 min for characterization and analysis of the exfoliated flakes.

### Graphene characterization

Various characterization tools were employed to evaluate the quality of the exfoliated graphene layers. As a first step, a scanning electron microscope (SEM) (Scios 2) manufactured by ThermoFisher Scientific was implemented to have an initial understanding of the particle size and morphology. The tool, also equipped with an energy dispersive spectroscopy (EDS) detector manufactured by Oxford Instrument (Ultim Max 40), was used for compositional analysis. The crystalline nature of the pristine graphite powder was assessed by X-ray diffraction (XRD, Bruker D8 Advance). Additionally, a Raman microscope (LabRAM Soleil) was used with an excitation laser of 532 nm. X-ray photoelectron spectroscopy (XPS) analysis was performed using a PHI 5000 VersaProbe Scanning X-ray Photoelectron Spectrometer. The system was equipped with a monochromated Al X-ray source (1486.6 eV) which was used as a probe for the experiments. Further, transmission electron microscope (TEM) images were collected using a Titan G2 model (ThermoFisher Scientific) to evaluate the thin flakes. Atomic force microscopy (AFM) was also used to analyze the thickness of the exfoliated thin layers. The AFM measurements were conducted in tapping mode using a Jupiter-XR system manufactured by Asylum Research Oxford Instruments. The scan rate was set to 1 Hz, with a high setpoint of ˂ 500 mV. The absorption study of the solutions was carried out in a PerkinElmer LAMBDA 1050 + UV–Vis–NIR spectrometer.

## Results and discussion

### Graphene exfoliation by probe sonication

**Figure 1:**
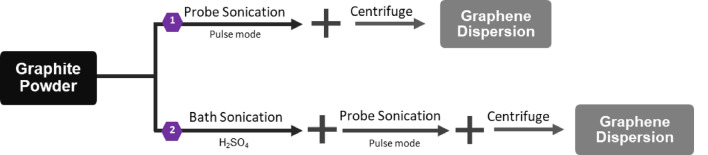
Graphene exfoliation approaches as implemented in this study.

Figure 1 depicts the methodology followed in this work to exfoliate graphene flakes from graphite powder. The purity and crystallinity of the as received graphite powder was evaluated by SEM, EDS, XRD, and Raman spectroscopy (see supplementary Fig. S1). Probe (ultrasonic tip) sonication was chosen due to its higher production rates of layered materials compared to methods like bath sonication^40^.

Probe sonication was performed in continuous and pulse modes. Pulse mode was found to be a more efficient exfoliation method to produce graphene flakes compared to continuous sonication. The continuous mode quickly raised the temperature of the sample dispersion, and it was difficult to stabilize even with ice bath. Controlling overheating is crucial to avoid chemical degradation and reduced concentration of the nanosheets^40^. On the contrary, the pulse mode can reduce sample heating if proper cycle times are used. It is worth mentioning that the probe sonication process also produces strong convection of the solution/medium. Since we have been using a lab scale beaker in our experiments, the observed convection is strong enough to distribute the temperature rise (produced by the probe tip) within seconds throughout the solution/medium. The probe induced temperature rise was then mitigated by keeping the dispersion in ice bath, replenishing the ice every two hours. Additionally, air flow at ~ 19 °C and 0.5 m/s was kept during the process as a further measure to keep the temperature rise in check. Hence, in the current study pulse mode with proper amplitude and cycle was implemented. Gradually, intermediate steps of acid treatment and centrifuging were integrated.

An initial dispersion was prepared with 1 wt% in 150 ml of deionized (DI) water. During sonication, the probe is in direct contact with the sample where it utilizes ultrasound energy. This ultrasonic agitation creates energetic cavitation bubbles due to the abrupt pressure fluctuations from the creation and bursting of these small bubbles^51^. Generating shock waves, liquid jets from bubble implosion and rapid oscillating forces all add to the powerful shear forces that proliferate layer delamination. It is also seen that within the cavitation zone/under the probe the bulk graphite flake layers were subject to layer tearing^47^. Therefore, ultrasonic cavitation can be used to physically exfoliate 2D materials by breaking the bulk lamellar materials into thinner flakes, which is the main mechanism for exfoliating graphene. After sonicating the sample for a specific time in pulse mode, the dispersion was left unperturbed during periods of time to separate the graphene flakes via sedimentation. Drops from the top portion of the dispersion were collected after 1, 24, and 94 h, drop-casted on precleaned Si substrates, and dried on a hot plate at 100 °C. The samples are named 1hr_sed, 24hr_sed and 94hr_sed, respectively. Once dried, the samples were investigated by SEM (see supplementary Fig. S2).

**Figure 2:**
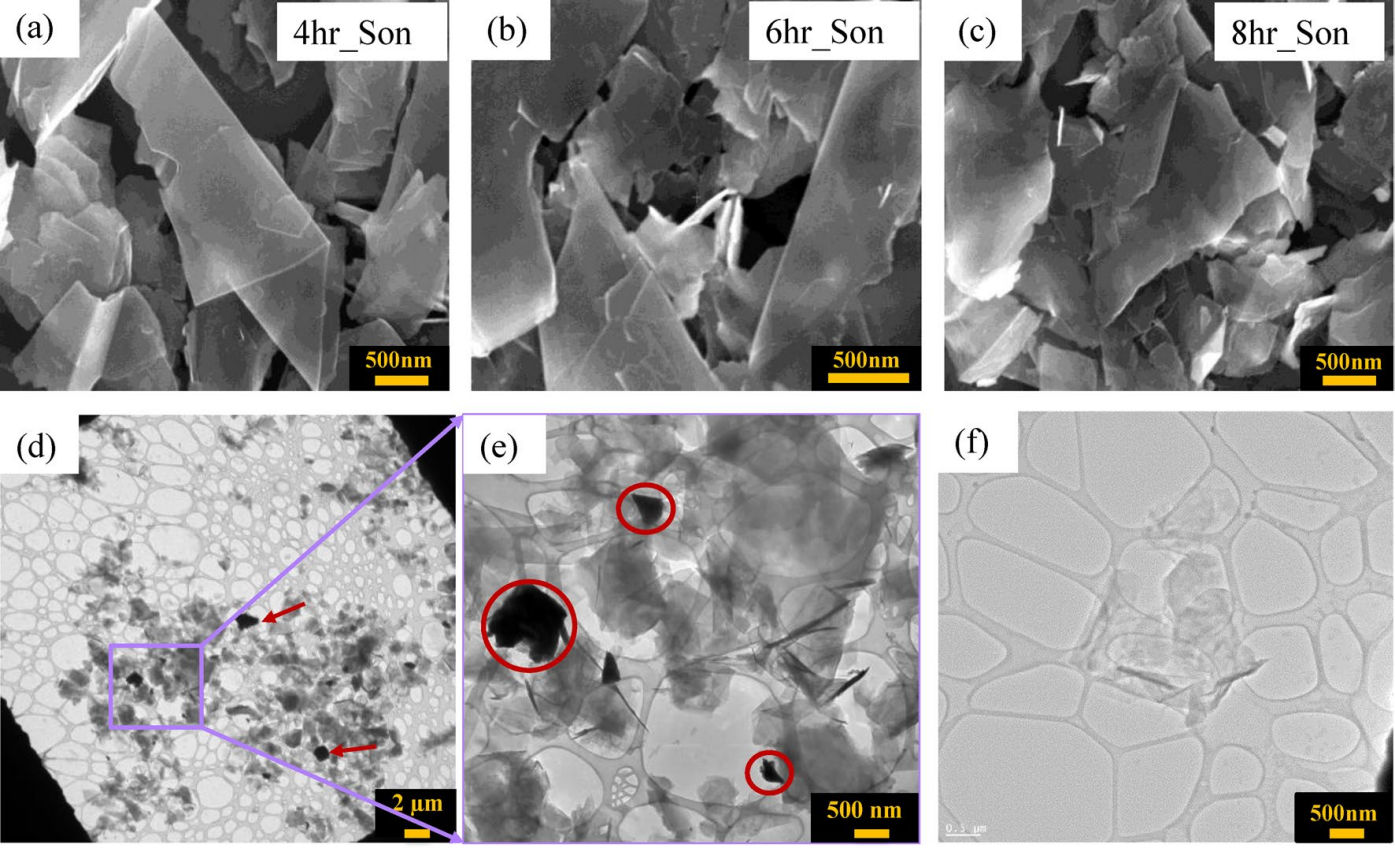
SEM images of the exfoliated graphene flakes of the samples were taken at different sonication times of (**a**) 4 h, (**b**) 6 h and (**c**) 8 h. Bright field TEM images of graphene flakes prepared using the probe sonication method. (**d**) Low magnification TEM image and a zoomed-in (**e**) TEM image. The thicker flakes appear darker compared to the thinner flakes and are shown by the arrows and encircled. (**f**) TEM image of the thin flakes indicating the presence of a few layers of graphene.

Afterward, the supernatant was further subjected to sonication with the previous parameters. Every two hours, the sonication was stopped, the dispersion was sedimented for an hour, and a sample was collected from the top portion for characterization. These samples are denoted as 4hr_son, 6hr_son, and 8hr_son, as shown in Fig. 2a–c respectively.

Further, a TEM sample was prepared from the 8hr_son sample. Figure 2d–f clearly indicate the widespread thin flakes of graphene with few microns in size. In addition to the thin flakes, a few thicker flakes are also observed, where the thicker flakes appear darker compared to the thinner flakes. Even though sedimentation was employed, a greater separating force was thus required. The images suggest the importance of implementing centrifuging to separate the bigger/thicker particles from the thinner flakes.

The impact of centrifuging was further evaluated following the probe sonication process. Probe sonication with higher sonication time (8hr_son) generates high number of defects in the graphene dispersion even though thinner flakes are created. This is in accordance with previous studies^45,52^ and so a trade-off between the sonication time and flakes with lesser defects must be made. In our study, a sonication time of 6 h was considered for further experiments (see supplementary Fig. S3) and the impact of centrifuging time was evaluated (see supplementary Table S1). The obtained SEM images of the flakes at each centrifuging interval showed less thicker flakes as centrifuge time was increased, see supplementary Fig. S4. Even though SEM imaging can only provide information on local scenarios, these results were expected and proven by the numerous literatures on size selection or liquid cascade centrifugation^53–55^. Therefore, for effective exfoliation and decantation, 1 h centrifuging was sufficient to separate the bigger particles from the smaller ones for coherent characterization.

### Acidic pretreatment

**Figure 3:**
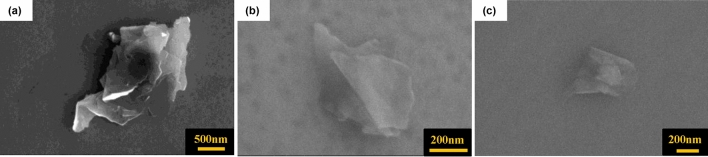
SEM images of graphene flakes subjected to acid pretreatment of (**a**) 0 M, (**b**) 3 M and (**c**) 5 M concentration of H_2_SO_4_.

We evaluated the impact of acid pretreatment on the exfoliation yield of graphene by performing bath sonication using different concentrations of H_2_SO_4_. The ionic size of sulfate ion is 0.46 nm, which is comparable to the graphite interlayer spacing (0.335 nm). This makes the sulfate ions a favorable candidate for the intercalation process for effective exfoliation of the graphene layers^56,57^. Sulfate-based ions are also used during anodic exfoliation of graphene from graphite^58^. Therefore, H_2_SO_4_ is chosen as the most appropriate pretreatment medium allowing sulfate ions intercalation between the graphene sheets and assisting in the exfoliation. The obtained acid pre-treated solution was rinsed with DI, filtered, and dried. The dried powder was resuspended in DI for further sonication following the parameters discussed above. After centrifugation, the samples were analyzed by SEM. Figure 3 depicts SEM images of the flakes exfoliated using 0 M (control; Fig. 3a), 3 M (Fig. 3b), and 5 M (Fig. 3c) H_2_SO_4_ solutions. Here, it is important to point out that during imaging we noticed that it was easier to find separated flakes in the pre-treated samples, suggesting an increased yield. However, the lateral size of the flakes was consistently reduced when increased H_2_SO_4_ concentration was used (Fig. 3).

**Figure 4:**
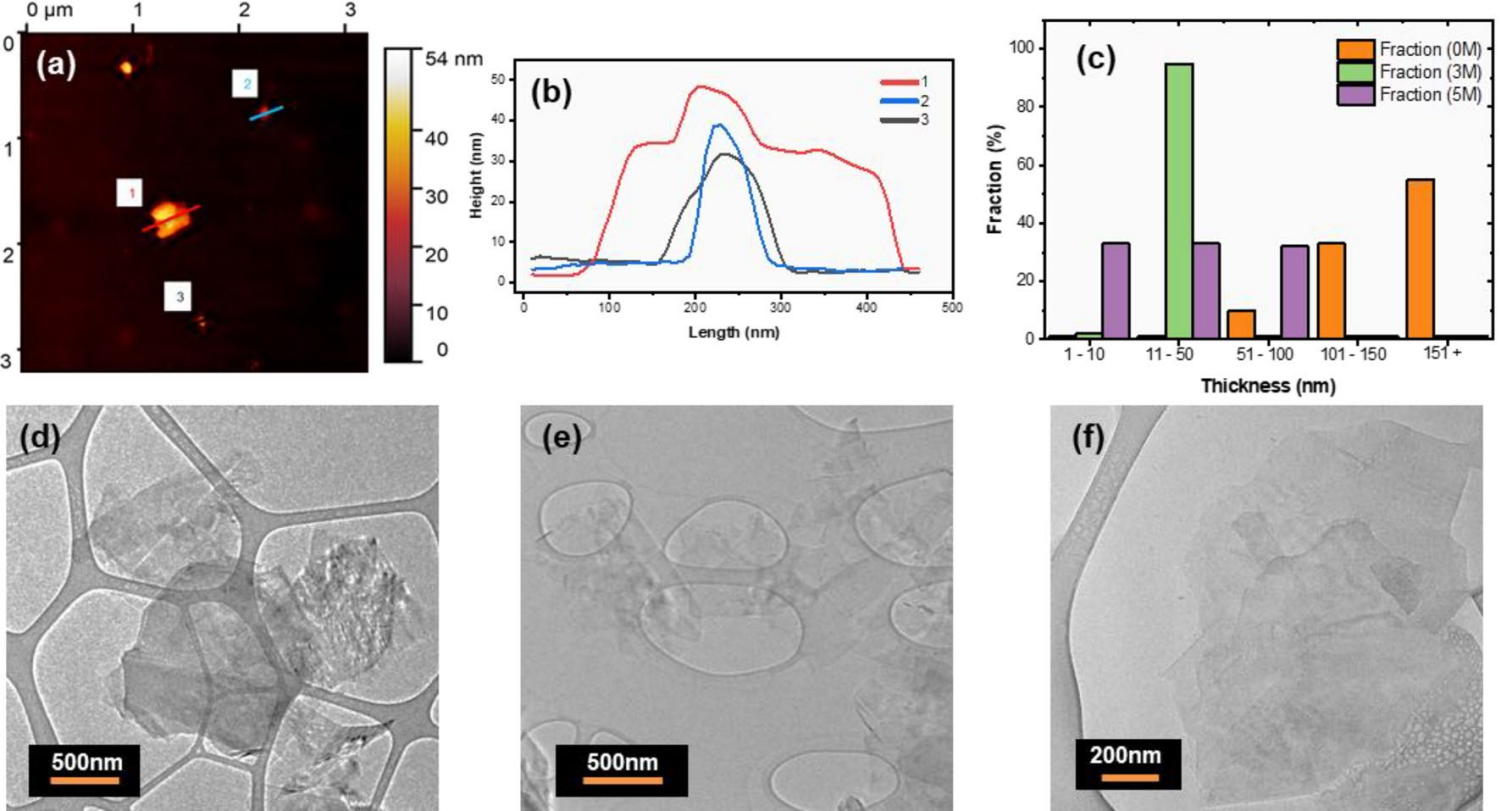
(**a**) AFM image of graphene flakes on silicon dioxide substrate, (**b**) and its respective height profile. (**c**) Statistical thickness analysis of the graphene flakes observed by AFM and calculated using Gwyddion software. Approximately ten flakes were measured for each sample. TEM images of thin flakes were taken from the (**d**) 0 M, (**e**) 3 M and (**f**) 5 M pre-treated samples.

The topography of the graphene sheets was investigated by AFM by depositing the graphene flakes on a heated SiO_2_ substrate. The thickness distribution was calculated using the height profiles obtained from the AFM images of the graphene flakes, as seen in Fig. 4a,b. It is to be noted that in addition to the stacking of the graphene sheets, under extreme conditions, some flakes could also bend. This could lead to a false height profile due to the resolution limit of the AFM tool. For the accurate height measurement as in the case of Fig. 4c, individual isolated flakes were considered. From each sample approximately ten flakes are measured, showing the statistical analysis of the flake thickness in Fig. 4c. The thinnest flakes (less than 10 nm) were seen in the sample pre-treated with 5 M H_2_SO_4_. TEM images obtained from pre-treated samples with different acidic concentrations are shown in Fig. 4d–f.

**Figure 5:**
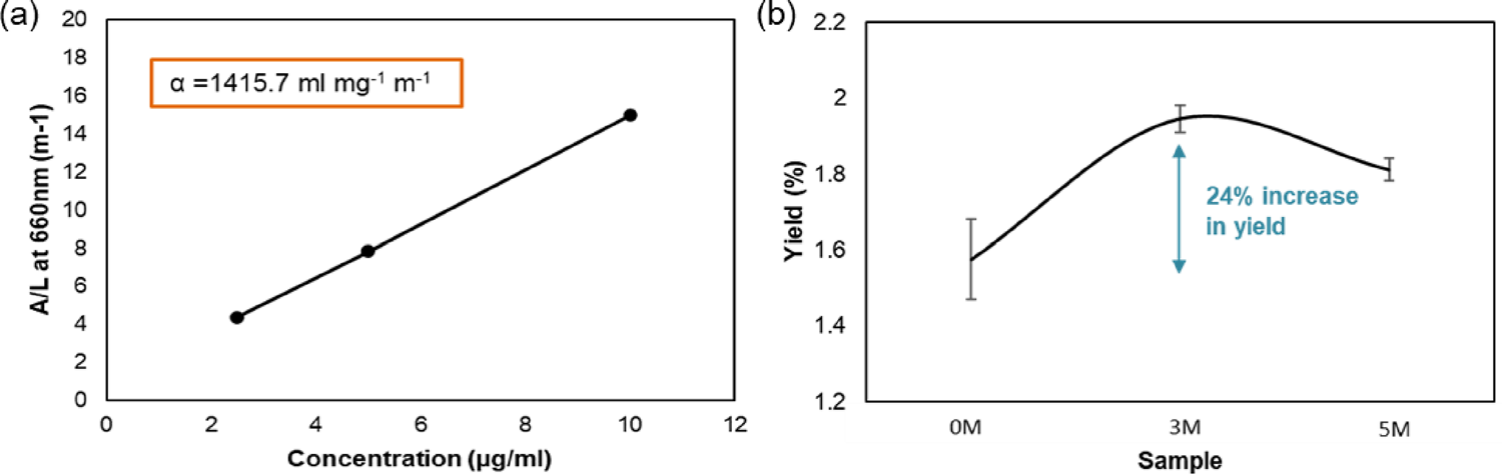
(**a**) Measurement of absorption coefficient of the graphene dispersions of the sonicated samples at known concentrations. (**b**) Yield comparison of the 0 M, 3 M, and 5 M graphene dispersions.

To further quantify graphene’s yield due to the pretreatment of the graphite powder, absorption study of the graphene solutions was performed. Figure 5a depicts the obtained absorption coefficient (α) (also named extinction coefficient^59^) from analyzing graphene’s baseline absorbance at 660 nm and applying Beer-Lambert’s law:

1 $$\frac{A}{L}=\alpha C$$

where A/L is absorbance per cell length, and C is the concentration.

The absorption coefficient, calculated to be 1415.7 ml mg^−1^ m^−1^, was within the range reported in the literature^60,61^. The pretreatment step has been found to increase the exfoliation yield by 24% as seen in Fig. 5b when comparing samples 0 M and 3 M. It is worth noting that the determined exfoliation yield is within the range that was expected with the liquid-phase exfoliation method^36,62^, see supplementary Table S2. The overall yield could be further increased by recycling the sediment after the centrifuging process that usually results in more graphene produced. Lastly, altering the parameters of the sonication process or careful choice of the starting graphite materials could further enhance the yield. The purity of the starting graphite powder is definitely very important. For example, functionalized form of graphite can significantly change the yield since the change of interlayer distance in the material will vary due to the presence of functional groups^2^ and this could impact the sulfate ion intercalation. Besides, the flake size is likely to impact as well. The sulfate ion intercalation starts from the edges which is the c-axis of the graphite structure. Coverage of the sulfate ion in the material/flake can be insignificant in the case of large flakes and this can decrease the graphene exfoliation. However, extensive parametric studies should be carried out in order to establish a flake size-dependent graphene exfoliation process.

**Figure 6:**
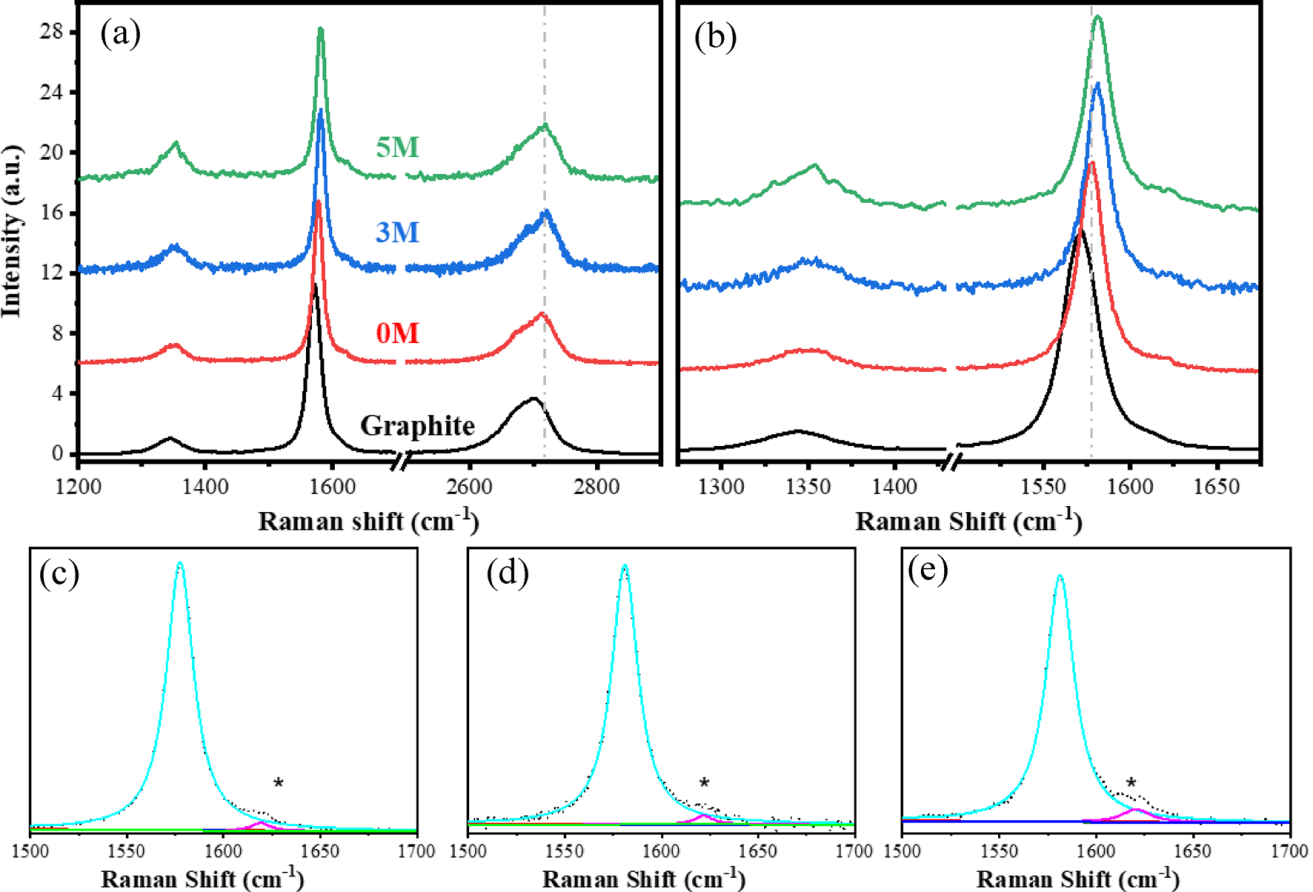
Raman spectra showing the evolution from graphite to graphene produced using different acid concentrations. (**a**) Raman spectra of graphite (black curve), and graphene under 0 M (red curve); 3 M (blue curve); and 5 M (green curve) acidic pretreatment; (**b**) corresponding spectra from the D and G region; Lorentzian curve fitting of the G (cyan curves) and D’ (magenta curve) bands of graphene produced using (**c**) 0 M; (**d**) 3 M; and (**e**) 5 M, acidic pretreatment. Vertical dashed lines and asterisks are guides to the eye.

To better understand the effect of the acidic pretreatment on the exfoliation process, the samples were analyzed by Raman spectroscopy. As shown in Fig. 6a, the Raman spectrum from graphite (black curve) and pre-treated dispersions at 0 M (red curve), 3 M (blue curve), and 5 M (green curve), presents a set of D, G and 2D bands typical of carbon layered materials. For graphite, the bands are centered around 1343 (D), 1571 (G), and 2700 (2D) cm^−1^^63^. In the case of pre-treated 0 M, 3 M, and 5 M samples, the D bands are localized at 1347, 1350, and 1349 cm^−1^, respectively. The G bands shift to 1577 (0 M), 1581 (3 M), and 1581.5 (5 M) cm^−1^ with respect to graphite (Fig. 6b)^64^. Similarly, the 2D bands upshift with respect to graphite to 2712 cm^−1^ for the 0 M sample and 2719 cm^−1^ for the 3 and 5 M, ones (Fig. 6a). These peak positions correspond to values reported for graphene^65–67^. Furthermore, an additional side peak near the G band emerged at 1619 cm^−1^ for the 0 M sample (Fig. 6c) and 1622 and 1621 for the 3 and 5 M samples, respectively (Fig. 6d,e). The rise of sub-peaks around 1617–1622 cm^−1^ has been reported for graphite-intercalated compounds (GICs) in stage-2 (one side of the carbon layer bound to the intercalant and the other side to another carbon layer) after acidic pretreatment^68,69^. This suggests the formation of GICs in our samples, although partial rather than an ideal full intercalation (Supplementary Fig. S5). GIC in general has been an interesting compound material. The formation of the GIC suggests the successful intercalation of the sulfate ions. Intercalation can expand the graphite flakes and reduce the interlayer attractive forces between the layers^70^. Due to the same reason, GIC has been used as an intermediate step for graphene exfoliation^71–73^. These studies suggest that GIC can be favorable for graphene exfoliation. Graphite intercalation compounds are generally achieved at either high concentration of sulfuric acid with other oxidizing agents, or with the help of electrochemical techniques^68,74^. Additionally, a chemical reaction (H_2_SO_4_ + C) may produce gaseous molecules such as, CO_2_, SO_2_ and H_2_O that can further push the layers to separate from each other^75^.

Further, the surface chemistry of the samples was evaluated to understand whether the addition of an acid pretreatment would result in the accumulation of functional groups attached to the graphene flakes. First, an EDS study revealed the presence of carbon, oxygen, and traces of sulfur in the 5 M sample. The presence of sulfur may result from trapped ions between restacked flakes^76^. Besides, a high oxygen content was found on specific regions of the flakes, indicating a partial oxidation of these (Supplementary Fig. S6). To confirm the increase of oxidation with the increase of acidic concentration, 0 M, 3 M and 5 M samples were analyzed by XPS.

**Figure 7:**
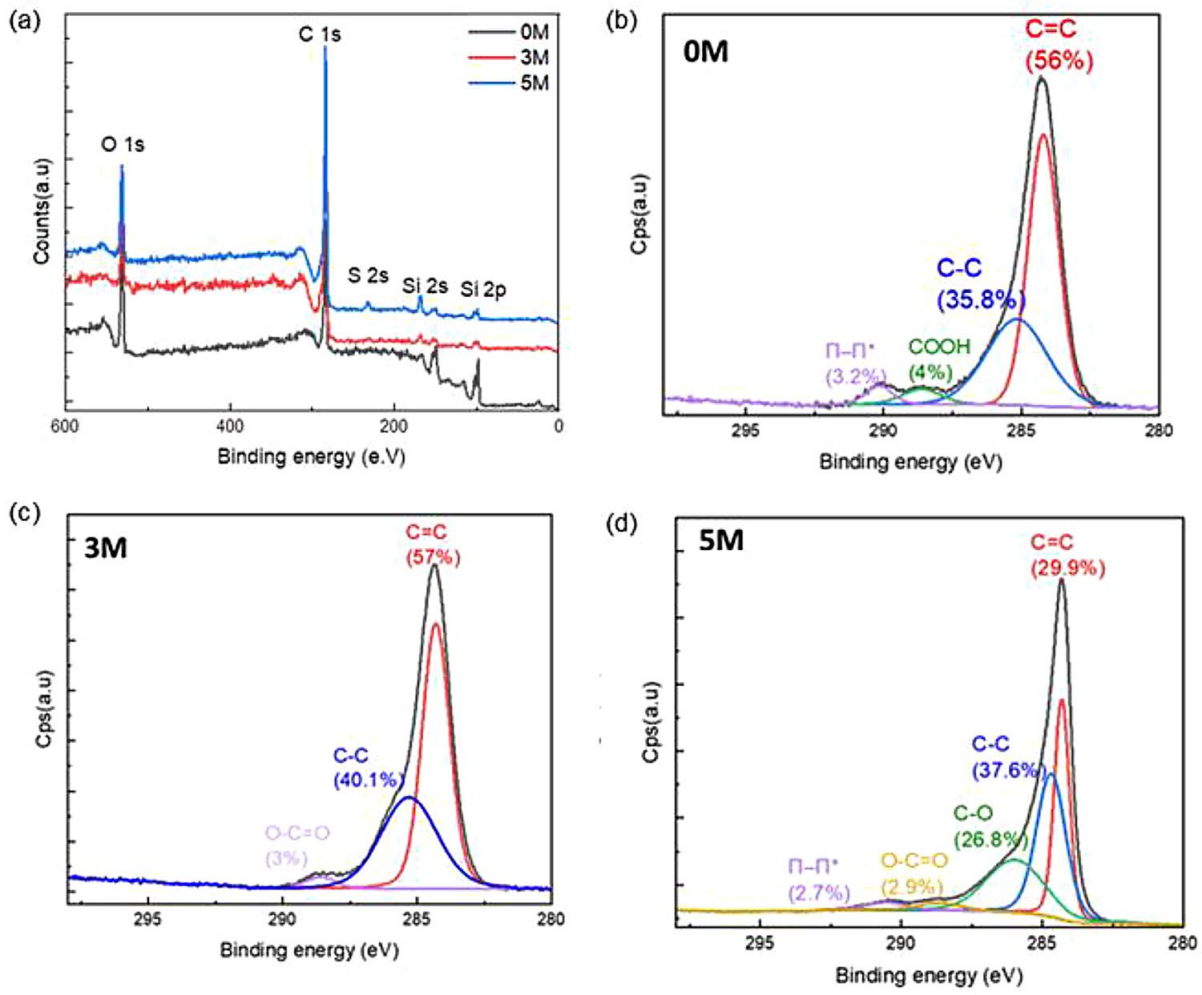
(**a**) XPS survey spectra of the exfoliated graphene flakes obtained at 0M, 3 M and 5 M bath sonication in H_2_SO_4_ electrolyte followed by probe sonication and subsequent centrifugation. Deconvolution of C 1s spectra of graphene obtained at (**b**) 0M, (**c**) 3 M and (**d**) 5 M in H_2_SO_4_ electrolyte after probe sonication and centrifugation.

The survey scans revealed the presence of three main components in all samples, which are denoted as: C1s at 286 eV, O1s at 532 eV and Si at 99 eV and 120 eV (Fig. 7a). The appearance of the Si peak is due to the fact that the flakes were prepared on a Si substrate and the X-ray beam diameter of the probe is ~ 100 µm, thus covering a wider area. The native oxide layer on top of the Si substrate can also contribute to the overall O1s peak. Additionally, there could be flakes with a thickness of less than 10 nm and since XPS information can come from ~ 10 nm of the sample, the substrate from below the thin flakes can also contribute to the overall O1s and Si peak. The 5M XPS survey spectrum (blue curve) showed the existence of sulfate ions (170 eV). The peaks at 532 eV confirm the presence of various oxygen functionalization in the carbon network structures caused by hydroxyl ions (OH^−^) during the chemical process. The high resolution C1s peak with different acid concentrations, elemental analysis, and corresponding peak deconvolution are shown in Fig. 7b–d. The fitted peaks occurring at about 284.5 and 285 eV are assigned to the unoxidized graphite carbon skeleton (C–C, C–H and C=C vacancies)^77^, while the peaks at around 286, 288 and 290 eV corresponds to hydroxyl or epoxide groups (C–OH or C–O–C), carboxyl group (C=O), and the pi–pi* peaks, respectively^78^. XPS analysis confirmed that the atomic concentration of oxidized carbon increases from 3% for the 3 M to 29.7% for the 5 M.

XPS measurements have been also used to evaluate the defect density in graphene^79^. According to several applied research^80^ and DFT simulations, the XPS peak at 285.2 eV indicates the existence of defects, while the peak broadening is directly related to the size of the defect^77,81–83^. Barinov et al.^84^ demonstrated that the line broadening and extra peaks are caused by the point defects present in the hexagonal carbon lattice. Additionally, as the defect density increases significantly, the form of the C1s line broadens significantly. The evolution of defect bands at the lower BE regime of C1s band requires an extra peak with BE of 285 eV to accommodate the substantial line broadening, and this peak was attributable to the existence of C adatoms or sp^3^-C bond over the surface. Additionally, the peak at 285 eV was attributed to defects such as nonconjugated carbon (nc-C) bonding in multi-walled CNTs and C–H bonding in hydrogenated graphite^85^.

As observed in Fig. 7, all the samples show peaks at 284 and 285 eV, indicating the existence of defects. The peak at about 284 eV was attributed to the presence of point defects that could result from C vacancy, with functional groups attached to the graphitic lattice. Additionally, the peak at 285 eV, was a result of the combination of the nc-C in the hexagonal lattice, which was due to a combination of C adatoms, non-aromatic C atoms and hydrogenation of carbon on the surface^86–89^.

As shown in Fig. 7c, the 3 M sample has a defect content of about 3%, while the 5 M sample presented a higher defect concentration, ~ 32% from which 5.6% come from vacancy defects and ~ 26.8% from nc-C defects. Moreover, the broadened shake-up (ShU) band at the high energy tail of the C1s spectrum was another indicator of the structurally superior graphitic material. The ShU high energy tail occurs due to the quick reaction of the electrons to the photoexcitation of core 1s electrons^90^. The quantity of C=C sp^2^ bonding in the hexagonal lattice determines the conjugation strength, which relates to the relative amplitude of this ShU signal. The relative area under the curve (AShU) was greater in 5 M sample, as shown in Fig. 7d. This confirms that the 3 M sample has fewer defects than the 5M one. In summary, the XPS spectrum analysis shows that the sample 3 M, developed with exfoliation of graphite in 3 M H_2_SO_4_, has the lowest total defect concentration whereas the sample 5M, pre-treated with higher acid concentration, has the largest vacancy and nc-C defects.

**Figure 8:**
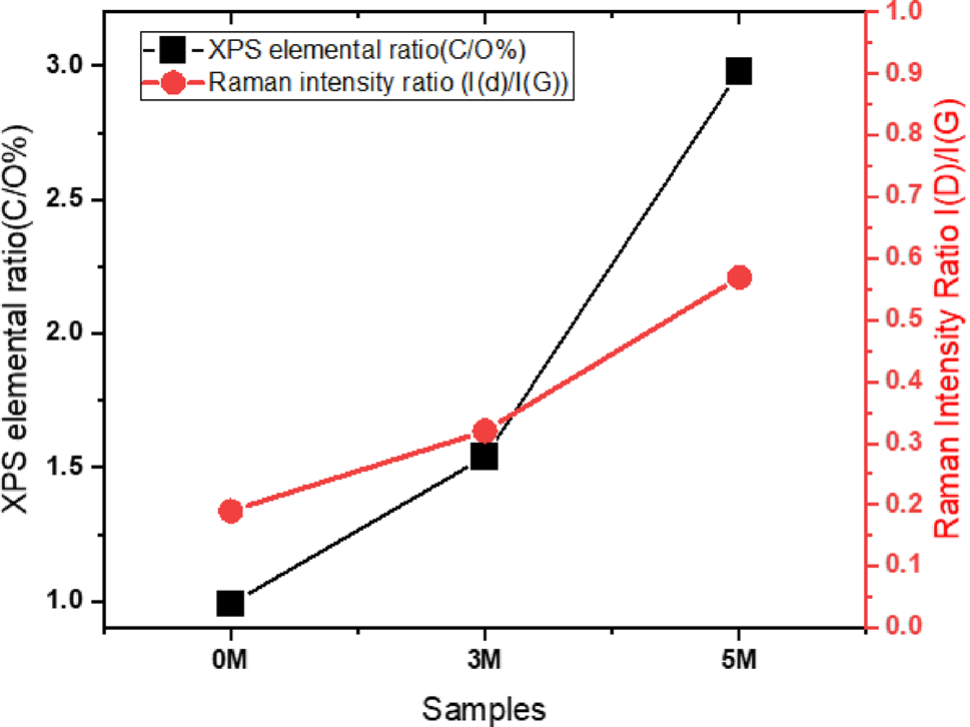
Change in the atomic ratio of carbon to oxygen determined from the XPS measurement and variations in I(D)/I(G) determined from Raman measurement.

Further, R_C/O_ was calculated from the XPS data and found that it was about 1 for the 0 M sample, which increased to about 1.54 for 3 M and 2.98 for the 5 M, in agreement with the I_D_/I_G_ value estimated from the Raman analysis (Fig. 8). The C/O atomic ratio and the D/G intensity ratio have a substantial correlation.

There are many techniques for the production and processing of graphene, but LPE is the simplest and most versatile method to produce stable dispersions. However, as Backes et al.^76^ mentioned, one of the main disadvantages of LPE is the choice of the liquid medium since this affects the pre- and post-processing conditions and final properties of the flakes, including rheology and stability. We have successfully demonstrated that pure water (DI) without the addition of any surfactant is a promising path for graphene exfoliation. We have also shown that the addition of an acid pretreatment is a simple and easy approach to increase the exfoliation yield of graphene. It is found to be very effective in overcoming the van der Waals forces and could generate thinner graphene flakes. As demonstrated, acid treatment with optimum molar concentration, such as 3 M or 5 M could introduce a sufficient number of functional groups to the graphene flakes, which can be further considered as a source material for the production of useful other graphene derivatives, such as holey graphene^2^.

The use of probe sonication allows the exfoliation of the graphene layers effectively. Increasing sonication time proves to be useful in thinning down the graphite flakes; however, it comes at the cost of reduction of the flake lateral sizes. This highlights the deteriorating side of probe sonication and limits its indefinite use in exfoliating graphene layers with high surface area. Despite the limitation, a trade-off could be made for an efficient use of the sonicating method. This could be beneficial for certain applications, such as controlling thermal reduction and pore creation or changing the inherent properties of the flakes.

## Conclusions

In this work, we have studied the exfoliation of graphene layers from graphite powder. Probe sonication was largely employed where parameters such as time, power, and centrifuge time were optimized to produce few-layer graphene flakes with size ˂ 1 µm. An intermediate sulfuric acid treatment was incorporated to increase the exfoliation yield. Increasing acidic concentrations produces thinner flakes but also increases the number of defects. Acidic pretreatment also introduces functional groups, which can be evident in the XPS studies. Thus, although sulfuric acid could help achieve thinner flakes, it inadvertently introduces certain functional groups and defects. Further, since the lateral size of the flakes decreased with increased acid concentration, we can conclude that the pretreatment with probe sonication not only promotes exfoliation between layers but also reduces their lateral size. This proves to be the major bottleneck for the full use of ultrasonic exfoliation, and a trade-off must be made between the flake size and thickness. Lastly, pretreatment with 3 M offers a 24% increase in graphene exfoliation yield with respect to the 0 M samples. The results also indicate that 3 M sample could be an ideal trade-off for obtaining thinner flakes with the highest yield and lower defect density. Further, the study and the understanding of the exfoliation process could be extended to other van der Waals materials for gaining thinner layers of 2D materials.

### Supplementary Information

Supplementary Information.

## Data Availability

The data that support the findings of this study are available from the corresponding author upon reasonable request.
