# Supplementary material for: Impact of probe sonication and sulfuric acid pretreatment on graphene exfoliation in water
Source: Sci Rep. 2023 Oct 28;13:18523. doi: 10.1038/s41598-023-45874-x (PMC10613256; doi:10.1038/s41598-023-45874-x)
Supplement: Supplementary file 1 — Supplementary Information. [file 41598_2023_45874_MOESM1_ESM.docx]

**Supplementary Notes**

**Impact of probe sonication and sulfuric acid pretreatment on graphene exfoliation in water**

Meriam Mohammedture^1, a,^ *, Nitul Rajput^1, a^, Ana Isabel Perez-Jimenez^1^, Zineb Matouk^1^, Shroq AlZadjali^1^, and Monserrat Gutierrez^1^

^1^Advanced Materials Research Center, Technology Innovation Institute, PO Box 9639, Masdar City, Abu Dhabi, UAE

*Corresponding author

E-mail: [Meriam.mohammedture@tii.ae](mailto:Meriam.mohammedture@tii.ae) / Tel: +971589440990

^a^These authors contributed equally.

**Figure S1. Characterization of the as received graphite powder.** **(a)** Low and **(b)** high magnification SEM images showing the morphology of the sample; **(c)** optical image of the graphite powder; **(d)** EDS spectrum of carbon, C, (20 keV, 0.1 nA); **(e)** XRD diffractogram indicating the crystallinity of the powder; (f) corresponding Raman spectrum of graphite characterized by the typical D, G and 2D bands centered around 1343 cm^-1^, 1571 cm^-1^, and 2700 cm^-1^, respectively.

**Figure S2**. SEM images of the exfoliated graphene flakes of the samples were taken at different sedimentation times.


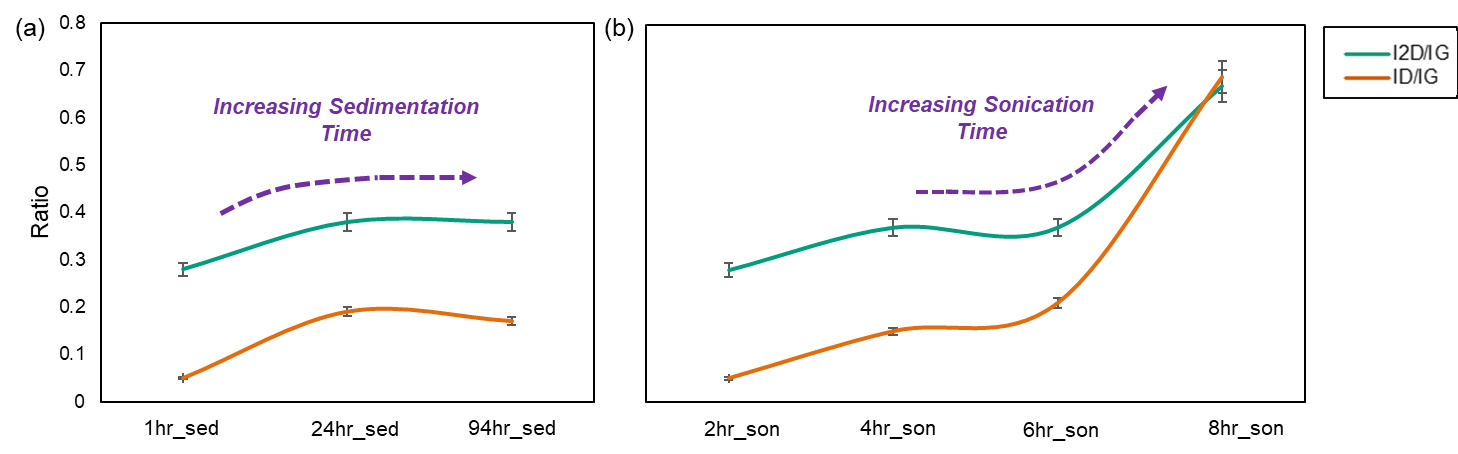


**Figure S3**. Raman peak analysis of the I(2D)/I(G) and I(D)/I(G) ratio (a) after increasing the sedimentation time from 1 to 94 hrs and, (b) after increasing sonication time up to 8 hours.

**Table S1** Parameters used for the analysis of the effect of centrifuge time.

| - | Concentration (mg/ml) | Volume (ml) | Sonication properties | Centrifuge (RPM)  19°C | *Centrifuge Time (mins)* |
| --- | --- | --- | --- | --- | --- |
| 2.4.1 | 0.1 | 175 | 57W  40% C  30% A | 5000 | *0* |
| 2.4.2 | 0.1 | 175 | 57W  40% C  30% A | 5000 | *15* |
| 2.4.3 | 0.1 | 175 | 57W  40% C  30% A | 5000 | *30* |
| 2.4.4 | 0.1 | 175 | 57W  40% C  30% A | 5000 | *60* |
| 2.4.5 | 0.1 | 175 | 57W  40% C  30% A | 5000 | *120* |
| 2.4.6 | 0.1 | 175 | 57W  40% C  30% A | 5000 | *180* |


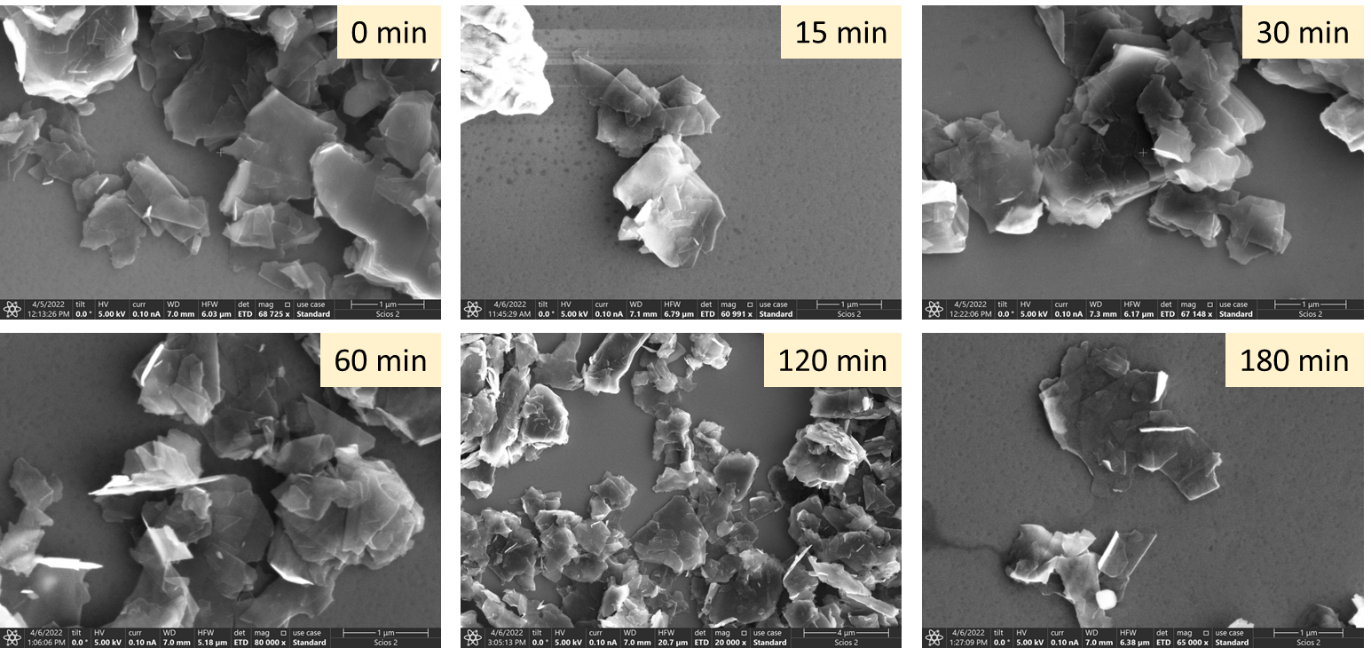


**Figure S4** SEM images of the samples centrifuged at different time.


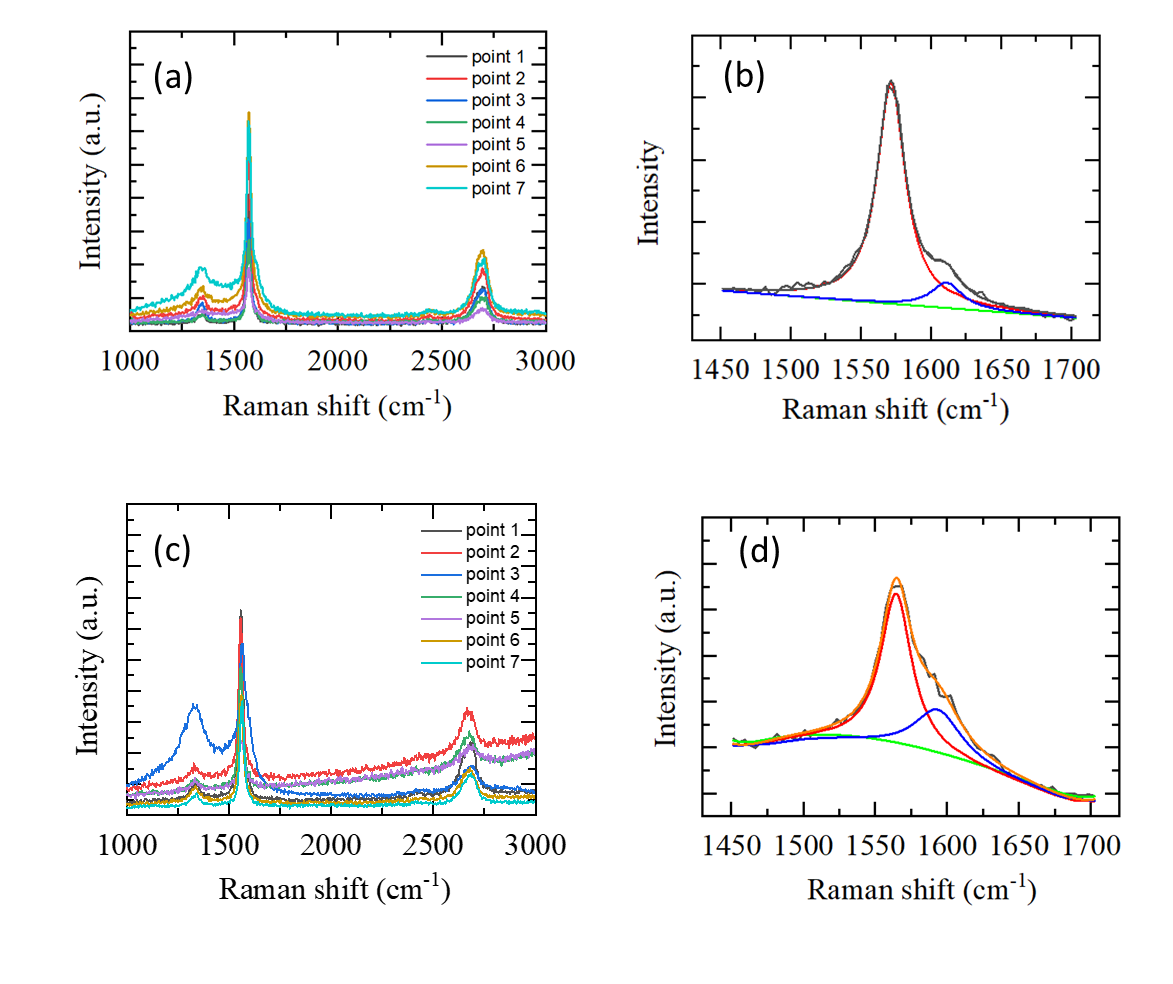
**Figure S5** Raman spectra acquired using the 532nm laser from different regions (points) of the graphite sample treated with H2SO4 concentration of (a) 3M and (c) 5M. Their deconvoluted G-peak fitted with Lorentz function are shown in (b) and (d) respectively.


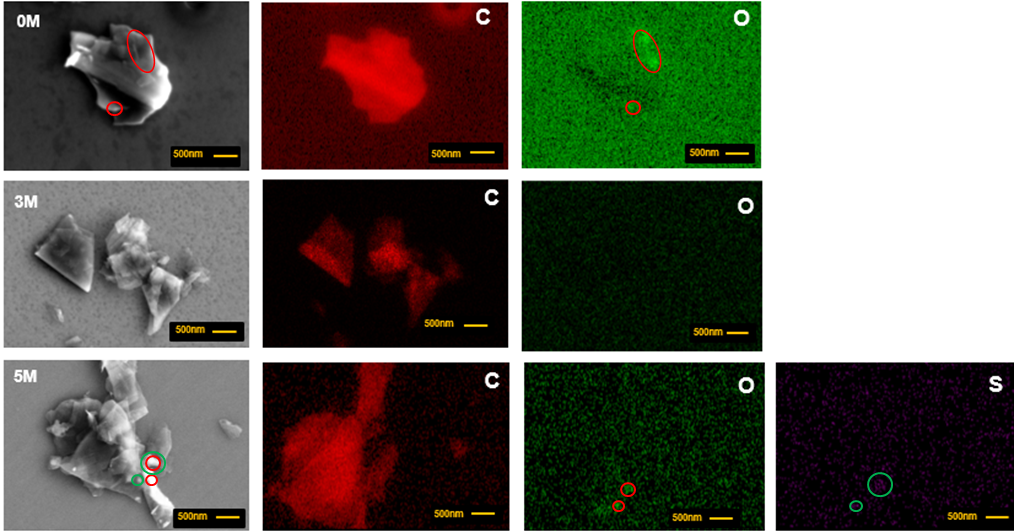


**Figure S6**. EDS images and analysis of the graphene flake surfaces to study the effect of adding sulfuric acid pre-treatment and comparison between the 0M, 3M, and 5M samples. The green circles denote areas detected with higher sulphur content. While red circles show more oxygen-enriched regions.

**Table S2** List of papers describing the liquid phase exfoliation of graphene with their exfoliation yields.

| **Exfoliation Method** | **Dispersions** | **Initial graphite concentration**  **(mg ml^-1^)** | **Exfoliation yield** | **Ref.** |
| --- | --- | --- | --- | --- |
| Shear mixer | Graphite in NMP, NaC or polymer polyvinyl alcohol | 1 – 100 | < 0.1% | [1] |
| Magnetic stirrer | Potassium graphite in THF | 6 | 4% | [2] |
| Ultrasound bath cleaner | Graphite in non-ionic and ionic aqueous surfactant solution | 100 | 1mg ml^-1^ / ̴1% | [3] |
| Bath sonication | Graphite in NMP | 2 – 8 x 10^-3^ | 1 wt% | [4] |
| Bath sonication | Graphite in NMP | 3.3 | 4wt% | [5] |
| Tip sonication | Graphite in NMP under azobenzene molecules irradiated with UV light | 10 | 1.1 % | [6] |
| Bath sonication | Graphite in NMP and docosanoic acid | - | 1.6% | [7] |
| Shear mixer | KMnO_4_ and H_2_SO_4_ pretreated graphite mixed with NaOH | 50 | 5wt% | [8] |
| Hydrodynamic cavitation | Graphite in DI water and Sodium Cholate | 50 | 2.5g / ̴12.5% | [9] |
| Bath sonication | Graphite in NMP and n-octylbenzene | 10 | 250µg/ml / ̴ 1.9% | [10] |
| *Probe sonication* | *Graphite in water with H_2_SO_4_ pretreatment* | *1* | *2%* | ***Our work*** |

**References:**

[1] K.R. Paton, E. Varrla, C. Backes, R.J. Smith, U. Khan, A. O’Neill, C. Boland, M. Lotya, O.M. Istrate, P. King, T. Higgins, S. Barwich, P. May, P. Puczkarski, I. Ahmed, M. Moebius, H. Pettersson, E. Long, J. Coelho, S.E. O’Brien, E.K. McGuire, B.M. Sanchez, G.S. Duesberg, N. McEvoy, T.J. Pennycook, C. Downing, A. Crossley, V. Nicolosi, J.N. Coleman, Scalable production of large quantities of defect-free few-layer graphene by shear exfoliation in liquids, Nature Materials, 13 (2014) 624-630.

[2] G. Bepete, E. Anglaret, L. Ortolani, V. Morandi, K. Huang, A. Pénicaud, C. Drummond, Surfactant-free single-layer graphene in water, Nature Chemistry, 9 (2017) 347-352.

[3] L. Guardia, M.J. Fernández-Merino, J.I. Paredes, P. Solís-Fernández, S. Villar-Rodil, A. Martínez-Alonso, J.M.D. Tascón, High-throughput production of pristine graphene in an aqueous dispersion assisted by non-ionic surfactants, Carbon, 49 (2011) 1653-1662.

[4] Y. Hernandez, V. Nicolosi, M. Lotya, F.M. Blighe, Z. Sun, S. De, I.T. McGovern, B. Holland, M. Byrne, Y.K. Gun'Ko, J.J. Boland, P. Niraj, G. Duesberg, S. Krishnamurthy, R. Goodhue, J. Hutchison, V. Scardaci, A.C. Ferrari, J.N. Coleman, High-yield production of graphene by liquid-phase exfoliation of graphite, Nature Nanotechnology, 3 (2008) 563-568.

[5] U. Khan, A. O'Neill, M. Lotya, S. De, J.N. Coleman, High-Concentration Solvent Exfoliation of Graphene, Small, 6 (2010) 864-871.

[6] M. Döbbelin, A. Ciesielski, S. Haar, S. Osella, M. Bruna, A. Minoia, L. Grisanti, T. Mosciatti, F. Richard, E.A. Prasetyanto, L. De Cola, V. Palermo, R. Mazzaro, V. Morandi, R. Lazzaroni, A.C. Ferrari, D. Beljonne, P. Samorì, Light-enhanced liquid-phase exfoliation and current photoswitching in graphene–azobenzene composites, Nature Communications, 7 (2016) 11090.

[7] S. Haar, M. Bruna, J.X. Lian, F. Tomarchio, Y. Olivier, R. Mazzaro, V. Morandi, J. Moran, A.C. Ferrari, D. Beljonne, A. Ciesielski, P. Samorì, Liquid-Phase Exfoliation of Graphite into Single- and Few-Layer Graphene with α-Functionalized Alkanes, The Journal of Physical Chemistry Letters, 7 (2016) 2714-2721.

[8] L. Dong, Z. Chen, X. Zhao, J. Ma, S. Lin, M. Li, Y. Bao, L. Chu, K. Leng, H. Lu, K.P. Loh, A non-dispersion strategy for large-scale production of ultra-high concentration graphene slurries in water, Nature Communications, 9 (2018) 76.

[9] X. Qiu, V. Bouchiat, D. Colombet, F. Ayela, Liquid-phase exfoliation of graphite into graphene nanosheets in a hydrocavitating ‘lab-on-a-chip’, RSC Advances, 9 (2019) 3232-3238.

[10] S. Haar, M. El Gemayel, Y. Shin, G. Melinte, M.A. Squillaci, O. Ersen, C. Casiraghi, A. Ciesielski, P. Samorì, Enhancing the Liquid-Phase Exfoliation of Graphene in Organic Solvents upon Addition of n-Octylbenzene, Scientific Reports, 5 (2015) 16684.
